# Scarlet Fever

**Published:** 1885-06

**Authors:** 


### The Treatment of Scarlet Fever by Scalded Oat-Meal.

As is very well known, the process of desquamation which follows scarlet fever varies very much in different individuals; sometimes it is accomplished by particles so fine as to be hardly perceptible, and these are a very frequent and certain source of contagion by means of clothes and otherwise, much more so than the scales as ordinarily thrown off. It is evident that this being the fact, it must be much more difficult to prevent a contact and contagion with these fine, almost imperceptible scales which are floating in the atmosphere, than where desquamation occurs in large patches of skin. To obviate this danger, Mr. George Smith, of Somerset, England (*Bristol Med. Chirurgical Journal*, Dec., '84), states that he has for several years been in the habit of having his patients well sponged over the surface of their bodies, commencing, as a rule, about a week after the appearance of the eruption, and continuing the process until desquamation is complete, with a mixture of one ounce of oat-meal to a pint of boiling water. The solution to be made fresh every day, and used tepid, or at such a temperature as may be comfortably borne by the back of a finger. His reason for using this particular combination is that the gluten in it sticks the scales to each other and to the surface of the body, thus allowing of their being removed from one sponging to another without the ordinary risk of infecting either atmosphere or clothes, and thus greatly lessening the risk of spreading the disease.

Secondly, the gluten fills up the cracks of the new skin and protects it from cold, as patch after patch of it becomes bare, and it thus, to say the least, greatly lessens the risk of the dropsy which so often follows upon this disease.

### Cheese and Ensilage.

Professor Williams, in 'The Chemistry of Cookery,' explains the reason of the indigestibility of cheese, as it is ordinarily cooked, by showing that casein dissolves neither in water, nor in alcohol, nor in weak acids ; but that it is readily dissoluble in alkalies ; and he proves that, in order to render it easily digestible, it is necessary to add to it sufficient alkali (carbonate of potash) to neutralize the acid it contains, to re-convert the casein into its soluble form, such as exists in milk. What applies to animal casein applies also to legumin, which Liebig calls vegetable casein. By boiling vegetables in water, and then throwing away the liquor, we get rid of some of the potash which is needful for the effectual digestion of those vegetables. Mr. Williams therefore advocates the addition of small quantities of bicarbonate of potash to such leguminous dishes as pea-pudding and lentil soup, especially when we use dried vegetables in their preparation. He also suggests that ensilage may be advantageously applied to food intended for consumption by human beings as well as to that designed for animals. Ensilage is a process of slow vegetable cookery—a digestion or maceration of fibrous vegetables in their own juices, until the fibre is loosened, rendered softer and more digestible, and partially converted into dextrine and sugar. And it is practically by ensilage that the hard Jersey pear, which we gather when fully grown, becomes in a month or two luscious and sweet. The process in this case is materially hastened and helped by the action of the natural acid of the fruit. In the case of sauerkraut the process is assisted by the artificial addition of acetic acid. Mr. Williams is of opinion that by using ensilage we may advantageously extend the sources of our food supply. Swedes and mangel-wurzels will become delicate diet for invalids ; and some day, perchance, it will be found possible to make good biscuit and pastry out of a mixture of sawdust with a little leguminous flour.—*St. James' Gazette.*

